# Is there a direct role for erythrocytes in the immune response?

**DOI:** 10.1186/1297-9716-42-89

**Published:** 2011-07-29

**Authors:** Davinia Morera, Simon A MacKenzie

**Affiliations:** 1 Institute of Biotechnology and Biomedicine, Universitat Autònoma de Barcelona, 08193 Barcelona, Spain

## Abstract

Erythrocytes are highly abundant circulating cells in the vertebrates, which, with the notable exception of mammals, remain nucleated throughout the entire life cycle. The major function associated with these cells is respiratory gas exchange however other functions including interaction with the immune system have been attributed to these cells. Many viral, prokaryotic and eukaryotic pathogens directly target this cell type and across the vertebrate group a significant number of related pathologies have been reported. Across the primary literature mechanisms of interaction, invasion and replication between viruses and erythrocytes have been well described however the functional response of the erythrocyte has been poorly studied. A fragmented series of reports spanning the vertebrates suggests that these cells are capable of functional responses to viral infection. In contrast, in-depth proteomic studies using human erythrocytes have strongly progressed throughout the past decade providing a rich source of information related to protein expression and potential function. Furthermore information at the gene expression level is becoming available. Here we provide a review of erythrocyte-pathogen interactions, erythrocyte functions in immunity and propose in light of recent -omics research that the nucleated erythrocytes may have a direct role in the immune response.

## Table of contents

1. Introduction

2. Erythrocyte-pathogen interaction

3. Immunity

4. Insights from genomic and proteomic studies

5. Future directions

6. Competing interests

7. Authors' contributions

8. Acknowledgements

9. References

## 1. Introduction

In all vertebrates, blood is composed of cells and plasma protein (90% in volume is water). By far the most abundant cell type in circulation is the erythrocyte, present in the nucleated form in the majority of vertebrates with the notable exception of the mammals. All vertebrates also have distinct populations of circulating leukocytes and non-mammalian vertebrates have nucleated thrombocytes instead of platelets. Erythrocytes are generally characterized as oval in shape and their characteristic red color is due to the respiratory globin pigments including the hemoglobins, the most abundant protein in these cells. All non-mammalian (birds, reptiles, amphibians and fish) erythrocytes with a few isolated exceptions are nucleated and contain organelles in their cytoplasm [1]. Erythrocyte longevity varies across the major vertebrate groups where in humans the cellular half life of erythrocytes is about 120 days and is about 40, 600-800, 300-1400 and 80-500 days in birds, reptiles, amphibians and fish respectively [1-5]. Furthermore and with a direct relationship to longevity, different maturation states are observed in the circulating erythrocytes of non-mammalian species including; changes in cytoplasmatic shape, staining, nuclear size and chromatin density. Total RNA and organelles content follow an inverse relationship with cellular age where young red blood cells contain a higher total concentration of RNA and aging cells display a loss of cellular organelles including ribosomes and mitochondria [6]. Interestingly, in endothermic species cellular longevity is similar indicative of a parallel evolutionary trend toward respiratory specialization linked in parallel to increased metabolic demand. However in view of the vast diversity of vertebrate species where the ectotherms are in the majority, the fish alone represent > 28 000 species, and the scarcity of such studies this proposed linkage warrants further investigation. Erythrocyte numbers in blood of vertebrates range from 1 to 5 × 10^6^/mm^3 ^and follow a descending evolutionary scale where the fish have the lowest cell counts [1,7]. This relationship can be further expanded including metabolic activity where highly active fish species, for example tuna, have higher numbers of circulating erythrocytes and show facultative endothermy [8]. Thus within the vertebrate group the erythrocytes appear to have significant differences in terms of circulating numbers and longevity which may reflect the metabolic needs of the species. However such a sweeping statement will clearly requires an empirical approach as the majority of these observations are derived from a small and likely non-representative set of vertebrate species.

Hematopoiesis is defined as blood cell formation from multi-potent stem cells that are characterized by self-renewal and the potential to differentiate into a mature set of distinct cellular phenotypes. Hematopoiesis has been shown to maintain a set of conserved molecular pathways based upon a conserved group of hematopoietic transcription factors present from humans to fish [9,10]. Erythropoiesis and myelopoiesis share cellular precursors and the production of erythrocytes or myeloid cells is dependent upon the key transcription factors Gata1 and PU.1 [11]. The regulation of these two transcription factors in the zebrafish embryo is a critical step in myelo-erythroid lineage commitment where Gata1 is required for erythroid cell differentiation and PU.1 for myelopoiesis [11-13]. Thus the molecular mechanisms underpinning hematopoiesis in fish and mammals appear to be remarkably conserved [14].

The principal function associated to the erythrocytes is oxygen and carbon dioxide transport. In addition to this gas exchange function, they also play a role in homeostasis protecting against oxidative damage and regulating blood flow distribution in skeletal muscle [15-17]. Other potential functions (Table 1) [18-44] have also been attributed to the erythrocytes for example: human erythrocytes may play a role in modulating T cell proliferation and survival by enhancing cytokine secretion and induction of the IL2R thus modulating CD4+/8+ ratios [21,45,46]. Interestingly this finding was published almost twenty years ago in sheep [47]. A fragmented series of publications can be identified across the vertebrates in which erythrocyte functions appear to be intimately connected to a range of physiological processes. These studies have shown that the cellular machinery and related biological processes are present in erythrocytes for intracellular signalling [16,48], transcription [49], protein synthesis [50] and secretion [51]. Functional responses attributed include amongst others; hemoglobin derived anti-microbial responses [23], glycophorin A-mediated pathogen sink [52], eNOS-like protein and activity [48], specific HIV-1 binding [53], interferon-alpha mRNA induction [29], hormone binding [54] and CR1-dependent immune complex clearance [55]. Thus it would appear that diverse ranges of non-respiratory biological processes in erythrocytes are observed across the vertebrates.

**Table 1:** Erythrocyte functions in vertebrates.

Proposed functions	Mammals	Fish	Amphibian	Reptils	Birds
Gas exchange function	[18]	[18]	[18]	[18]	[18]
Sugar transport	[19]	[26]	[34]	[36]	[40]
Calcium homeostasis	[20]	[27]	[35]	[37]	[41]
Redox homeostasis	[17]	[28]	-	[38]	[42]
Cell proliferation	[21]	-	-	-	[43]
Antiviral response		[29]	-	-	-
*Antimicrobial activity*					
Immune complex	[22]	[30]	-	-	-
ROS production	[23]	[31]	-	-	-
Hemoglobin	[24]	[32]	-	[39]	-
Other related functions	[25]	[33]	-	-	[44]

Table reports functions associated to erythrocytes in mammals, fish, amphibian, reptiles and birds. References are numbered according to appearance.

## 2. Erythrocyte-pathogen interaction

There exists a broad spectrum of parasites that specifically invade the erythrocytes of vertebrates (Table 2) [53,56-66] causing a diverse set of negative consequences and disease in the host organism. The host environment is complex where a significant number of inter-related biological factors including both physical and biological barriers such as the skin and the mucosal immune system influence the ability of pathogens to invade the host. Once a pathogen has cleared the primary barriers and accesses the circulatory system the erythrocytes may become a primary target for pathogen dispersal/transport throughout the organism. However not all parasites may induce damage within the host. For example, bacteria of the *Anaplasmatacea *family form inclusions in the erythrocyte cytoplasm but have not been observed to cause disease or negative health effects either in the host organisms or in host erythrocytes [4]. A significant lack of knowledge exists about many of the erythrocyte-related pathologies at the level of host-pathogen recognition and the molecular mechanisms underpinning erythrocyte responses; the major exception being the biology of the *Plasmodium *species where more than 200 are known and almost half of these species target the lizards [65,67-69]. The importance to human health of this particular parasite has fueled much research that has uncovered a high degree of complexity related to host specificity, underlying molecular mechanisms and host response. We will not review the malaria literature as this has been extensively covered elsewhere [58,70]. Instead here we will focus upon the basic knowledge that supports an active role for the nucleated erythrocytes in vertebrate immunity.

**Table 2:** Parasites of vertebrate erythrocytes.

Family	Genera	Mammals	Fish	Amphibia	Reptils	Birds
Dactylosomatidae	Babesiosoma	[56]	[4]	[4]	[4]	[62]
Dactylosomatidae	Dactylosoma	-	[4]	[4]	[4]	-
Garniidae	Garnia	-	-	-	[4]	[63]
Garniidae	Progarnia	-	-	-	[4]	-
Garniidae	Saurocytozoon	-	-	-	[4]	-
Haemogregarinidae	Cyrilia	-	[4]	-	-	-
Haemogregarinidae	Desseria	-	[4]	-	-	-
Haemogregarinidae	Haemogregarina	[57]	[4]	[4]	[4]	[57]
Haemogregarinidae	Hemolivia	-	-	[4]	[4]	-
Haemogregarinidae	Hepatozoon	[57]	[4]	[4]	[4]	[57]
Haemogregarinidae	Karyolysus	-	-	[57]	[4]	-
Haemoproteidae	Haemoproteus	[57]	-	[4]	[4]	[64]
Haemoproteidae	Haemocystidium	-	-	-	[4]	-
Haemoproteidae	Simondia	-	-	-	[4]	-
Lankesterellidae	Lainsonia	-	-	-	[4]	-
Lankesterellidae	Lankesterella	-	-	[4]	[4]	[57]
Lankesterellidae	Schellackia	-	-	[4]	[4]	-
Plasmodiidae	Billbraya	-	-	-	[4]	-
Plasmodiidae	Mesnilium	-	[4]	-	-	-
Plasmodiidae	Plasmodium	[58]	-	-	[4]	[65]
Trypanosomatida	Sauroleishmania	-	-	-	[4]	-
						
Uncertain	Chelonoplasma	-	-	-	[4]	-
Uncertain	Cingula	-	-	-	[4]	-
Uncertain	Erythrocytonucleophaga	-	-	[4]	-	-
Uncertain	Globidiellum	-	[4]	-	-	-
Uncertain	Haematractidium	-	[4]	-	[4]	-
Uncertain	Haemohormidium	-	[4]	-	[4]	-
Uncertain	Sauromella	-	-	-	[4]	-
Unceratin	Tunetella	-	-	-	[4]	-
						
Anaplasmataceae	Aegyptianella	-	-	[4]	[4]	-
Anaplasmataceae	Bertarellia	-	-	[4]	[4]	-
Anaplasmataceae	Cytamoeba	-	-	[4]	[4]	-
Anaplasmataceae	Eperythrozoon	[59]	[4]	-	-	-
Anaplasmataceae	Haemobartonella	[59]	[4]	[4]	[4]	-
Bartonellaceae	Grahamella	-	-	-	[4]	-
						
Viral	Orthomyxoviridae Influenza A	[60,61]	-	-	-	[66]
Viral	Retroviridae Lentivirus (HIV-1)	[53]	-	-	-	-
Viral	Orthomyxoviridae Isavirus (ISAV)	-	[4]	-	-	-
Viral	unknown Immanoplasma*	-	[4]	-	-	-
Viral	unknown Pirhemocyton*	-	-	[4]	[4]	-
Viral	unknown Toddia*	-	[4]	[4]	[4]	-

Table reports parasitic genera infecting both nucleated and non-nucleated vertebrate erythrocytes. References are numbers according to bibliography listing.

*These genus names are not accepted viral genera, but were designated in the early 1900s based on erythrocyte inclusions thought to be caused by protozoan parasites. The inclusions were subsequently found to be associated with icosahedral virus particles considered most likely to be large DNA viruses, possibly in the family Iridoviridae, but the taxonomy has not been conclusively defined.

Different families of parasites invade erythrocytes both from mammals (enucleated) and non-mammals (nucleated). In 2000, Davies et al. presented an extensive list of intra-erythrocytic parasites compiled from mammals, birds, amphibian, reptiles and fishes [4]. Table 2 summarizes family and genera of parasites that infect mammalian and non-mammalian erythrocytes. Protozoan, prokaryotes and viral parasites are indicated in the table with others of uncertain taxonomic status or identity. Although birds, amphibian, reptiles and fish are all affected by different parasite genera, the reptiles have been shown to be the most highly affected group by the range of known parasites. This however may again be bias in view of the representation of the species studied and sampled. Rather than favoring infection of mammals or non-mammals, *Haemohormidium *and *Dactylosoma *are distinct in infecting the cold-blooded vertebrates; fish, reptiles and amphibians [4]. The interactions of viral particles with the host are determined by two major factors; host cell membrane proteins and the molecular determinants on the virion surface. The presence or absence of specific receptors on the cell membrane and their organization are key elements for virus invasion and the development of disease in the vertebrates. Virus-cell interaction is a central issue in animal/human health and the development of prophylactic medicines a key element in combating viral disease [71]. In 1979, infection and de novo synthesis of the influenza virus (A/FPV/Rostoch/34 (Havl N1) proteins in nucleated erythrocytes of birds was described for the first time [66]. Following this a significant body of studies using the influenza virus genera have been published describing in great detail erythrocyte-influenza virus interaction in a range of vertebrate groups including humans where the viral hemagglutinin glycoprotein (HA) has been shown to mediate specific recognition, binding and fusion between the virus and the target cell [60,61]. Surprisingly the response of erythrocytes to viral infection has been poorly studied especially in the nucleated erythrocytes of the non-mammalian vertebrates.

## 3. Immunity

Nelson (1953) described erythrocytes as directly participating in the immune complex reaction (bacteria, complement and antibody) and this specific binding suggested a central role for this cell type [72]. This interaction of erythrocytes with the immune complex has been repeatedly shown (reviewed in [55]). Fish and chickens erythrocytes have been shown to actively form rosettes to facilitate the clearance of pathogens by macrophages [73], and could produce cytokines or specific signalling molecules in response to binding [44,74]. In 1999, Bishlawy et al. hypothesized a relationship between erythrocytes, hemoglobin and the immune system suggesting an active role in the immune response to pathogens [75]. A supporting argument may be found in several studies of hemoglobin function. Hemoglobin is an important source of bioactive peptides that have been shown to participate in the innate immune response [23,24]. The antimicrobial activity of the respiratory globins is likely one of the most ancient anti-microbial mechanisms conserved across the invertebrates and vertebrates [76]. These respiratory protein-derived peptides exhibit antimicrobial activity against Gram-positive, Gram-negative bacteria and yeast [24,77]. Although little attention has been given to these observations it remains clear that vertebrate hemoglobins and associated molecules in invertebrate species have bactericidal properties and participate in the killing of invading microbes. Glycophorin-A (GYPA) is the most abundant glycoprotein on the mammalian erythrocyte cell surface (0.5-1 × 10^6 ^copies per cell) [78] and it has been hypothesized that GYPA plays a role in pathogen recognition potentially acting as a decoy receptor in enucleated mammalian erythrocytes. Thus GYPA would bind pathogens on the erythrocyte cell surface thereby sequestering invading organisms from important tissues potentially reducing pathogen load. It has been suggested that these pathogen bound to erythrocytes would be cleared by macrophages in the spleen [52]. Supporting observations for erythrocyte specific binding of "active" viral particles was recently demonstrated for HIV-1 in human erythrocytes [53]. In both fish and chicken, specific PRRs (pathogen recognition receptors) including members of the toll-like receptors (TLRs) and peptidoglycan recognition protein (PGRP) receptor families have been shown to be expressed in erythrocytes and ligand-specific engagement induces a physiological response that is stimulus-dependent [79,80]. Thus an active role in pathogen recognition must be considered and leads to the hypothesis that the presence of PRRs in non-mammalian erythrocytes and an associated pathogen associated molecular pattern (PAMP)-specific response strongly points toward a direct role for nucleated erythrocytes in the immune response.

The production of type I interferon is a well-characterized immune response that bridges the innate and acquired immune responses and has been shown in most vertebrate groups [81,82]. Workenhe and colleagues published the first study showing that erythrocytes could play an immunological role in virus response observing that nucleated erythrocytes from Atlantic salmon produced α-interferon in response to ISAV (infectious salmon anaemia virus) infection [29] however no more studies have been reported in this field or in other species. Studies in rainbow trout and chicken erythrocytes demonstrate that the stimulation of these cells with different PAMPs regulate the synthesis of different mRNAs of immune response related genes like TLRs, CCL4 or α-interferon [80].

## 4. Insights from genomic and proteomic studies

The emergence of high-throughput sequencing technologies both for genomic and proteomic studies has steadily been changing the molecular landscape of modern biological research. In the case of the erythrocytes, in human cells, significant advances have been made in characterizing the proteome of this enucleated cell in search of prognostic markers for disease. In 2002 a total of 102 proteins where identified by 2-D electrophoresis and MALDI-TOF analysis [83], this was rapidly followed in 2004 by the description of 181 protein sequences from the erythrocyte membrane and cytoplasm using ion trap mass spectrometry [84]. In 2005, 272 erythrocyte proteins were identified using a novel approach, 2-D nano-high performance liquid chromatography and electrospray ionization tandem mass spectrometry (2-D nano-HPLC-ESI-MS/MS) [85]. Following this trend over the past decade a total of 1989 non-redundant human erythrocyte proteins have been identified [86]. An important observation made from these studies is that all authors agree that human erythrocytes express proteins that are related to cellular defence, the immune response and potentially multiple other biological functions. This supports the hypothesis that the erythrocytes even in the mammalian enucleated form have the potential as described by the proteome to participate in the immune response.

**Figure 1:**
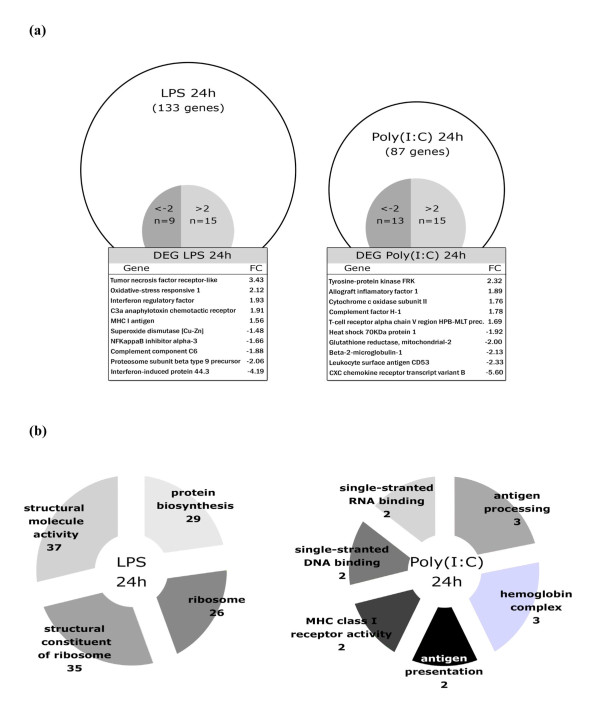
**Microarray analysis (SFA2.0 cDNA salmonid-specific array**. Accession number; GPL6154) in rainbow trout erythrocytes stimulated for 24 h with LPS or poly (I:C). (a) Total number of differential expressed genes (DEG) identified using a p-value of < 0.01. The total number of DEG with a fold change greater than 2 (FC > 2) (*t*-student, *p *< 0.01) are shown and selected DEG with relevance to immunity (*p *< 0.01) are shown. Results obtained from three independent experiments for each PAMP (five animals for each experiment). (b) Over representation of Gene Ontology functional categories. Categories were selected with Yates corrected Chi squared (*p *< 0.05). The number of DEG for each overrepresented category is shown.

At the level of the transcriptome human erythrocytes have been shown (Human Genome Focus GeneChip, *Affymetrix*) to contain multiple mRNAs with direct relevance to biological processes including signal transduction and the immune response [87]. Recently in fish, custom cDNA microarrays (16 K cGRASP) have been used in the rainbow trout (*Oncorhynchus mykiss*) to explore the potential for specific erythrocyte mRNAs as prognostic markers for stress. In this study genes related to stress physiology and the immune response were shown to be the most highly regulated under a heat stress experimental regime [88]. In line with these results, analysis of isolated trout erythrocytes challenged for 24 hours with either lipopolysaccharide (LPS) or poly (I:C), a double stranded RNA analog, can identify a PAMP-specific regulation of gene expression in erythrocytes (Figure 1a). Analysis of over-representation of Gene Ontology (GO) categories (Figure 1b) identified that LPS challenge strongly affected biological processes broadly related to protein synthesis where four differentially represented GO categories including protein biosynthesis, ribosome, structural constituent of ribosome and structural molecule activity were significantly different after challenge. Interestingly, the response to poly (I:C) was characterized as related to the immune response: GO categories related to antigen processing, antigen presentation and MHC class I receptor activity, moreover single-stranded RNA-binding, single-stranded DNA-binding and hemoglobin complex were up-regulated. These preliminary results highlight two important points; firstly, PAMP challenge induces a specific response at the level of the erythrocyte transcriptome and secondly this response is dependent upon the PAMP itself thus is likely to be PAMP-PRR specific.

## 5. Future directions

In conclusion, the increasing knowledge of parasite biology in parallel to a growing body of research effort aiming toward understanding host-pathogen interactions using the -omics technologies is opening a broad spectrum of potential avenues for investigation of vaccines for the control and prevention of disease. The contributions from both genomics and proteomics have provided significant advances toward the understanding of the vertebrate erythrocytes. Importantly across the entire vertebrate group several studies have now shown that the erythrocytes express proteins and mRNAs that are related to physiological processes that appear unrelated to the highly specialised role ascribed to these cells. Thus it may appear that an as yet unexplored series of potential functions for the erythrocytes may be present that in turn implies a more diverse physiological role for these abundant cells.

## 6. Competing interests

The authors declare that they have no competing interests.

## 7. Authors' contributions

DM performed experimentation, analysis and drafted the manuscript. SM conceived, designed and coordinated the study. Both authors read and approved the manuscript.
